# Fresh preservation of alfalfa sprouts and mushroom slices by soaking with thymol and resveratrol solutions

**DOI:** 10.1002/fsn3.458

**Published:** 2017-02-09

**Authors:** Li‐Jung Lai, Ju‐Min Chiu, Robin Y.‐Y. Chiou

**Affiliations:** ^1^ Department of Food ScienceNational Chiayi UniversityChiayiTaiwan; ^2^ Department of Food NutritionChung Hwa University of Medical TechnologyTainanTaiwan

**Keywords:** alfalfa sprouts, *Escherichia coli*, fresh preservation, resveratrol, *Staphylococcus aureus*, Thymol

## Abstract

The preservation of fresh produce served as salads through soaking with solutions containing naturally occurring phenolic ingredients is of merit. For a primary assay, thymol and resveratrol at 0–500 ppm were prepared and used to inhibit growth and survival of *Escherichia coli* and *Staphylococcus aureus*. Thymol and resveratrol exhibited potent inhibitory activities against the growth of both bacteria. For *S. aureus*, cells treated with thymol at 250 ppm or resveratrol at 500 ppm, the durations to achieve 3 log reduction (3LR) were 40 and 20 min, respectively. When the cells were treated with thymol combined with resveratrol, both at 250 ppm, the 3LR value was achieved in under 5 min**.** Synergistic antibacterial activity between thymol and resveratrol was apparent. The antibacterial and known health‐enhancing activities of resveratrol are of interest.

## 1 Introduction

The discovery and use of environmentally friendly, natural, and cost‐effective substances for vegetable cleaning, in particular for fresh produce served as salads, is the goal of multidisciplinary green chemists, related industries, and consumers. Thyme is commonly used in kitchens as a food seasoning herb and is a major source of thymol. Thyme is designated by the U.S. Food and Drug Administration (FDA) as generally recognized as safe (21 CFR 182. 10, 21 CFR182.20). In addition to thymol, several derived phenols of thymol are also listed as safe food additives by the FDA (21 CFF 172.515; synthetic flavoring substances and adjuvants). Thymol (C_10_H_14_O, 2‐isopropyl‐5‐methylphenol) is a natural monoterpene phenol derivative of cymene, commonly found in oil of thyme (*Thymus vulgaris*) and other sources, such as oregano, tangerine, and honey. After extraction and purification, it is a white crystalline substance bearing a pleasant aromatic odor and with strong antiseptic properties (Burt, 2004; Burt & Reinders, 2003; Burt, Vlielander, Haagsman, & Veldhuizen, 2005; Calo, Crandall, O'Bryan, & Ricke, 2015; Lu, Joerger, & Wu, 2014; Lu & Wu, 2010; Xu, Zhou, Ji, Pei, & Xu, 2008). Use of thymol as a cost‐effective antimicrobial for securing food safety and ensuring quality deserves further investigation.

Resveratrol (3,5,4′‐trihydroxy‐*trans*‐stilbene) is a member of the stilbene family of natural polyphenols. It was isolated and identified as a phytoalexin from grape vines after *Botrytis cinerea* infection (Lancake & Pryce, 1976). In general, the biosynthesis of resveratrol is induced in response to biotic or abiotic challenges in several food plants, such as grapes, various berries, and peanuts, as a secondary metabolite (Giovinazzo, Ingrosso, Paradiso, De Gara, & Santino, 2012; Wang et al., 2005). In recent years, novel biological activities of resveratrol have been demonstrated. Use of resveratrol as an ingredient applied for product formulation in the chemoprevention of chronic diseases, chemotherapy, healthcare functional foods, nutraceuticals, and cosmetics has attracted considerable interest from the academic and industry communities. No in‐depth studies into the antimicrobial nature of resveratrol have been undertaken; thus, food processing to combine the antimicrobial and health‐enhancing properties of resveratrol to guarantee food safety, hygiene quality, and health benefits is of novelty (Paulo, Ferreira, Gallardo, Queiroz, & Domingues, 2010; Paulo, Oleastro, Gallardo, Queiroz, & Domingues, 2011).

In recent years, attempts to use natural antimicrobials in food processing have expanded rapidly, mainly in response to the increasing demand for greener additives from consumers (Solorzano‐Santos & Miranda‐Novales, 2012). Over the last two decades, natural preservatives have begun to be used for practical applications, and some technologies have shown that the inactivation of microorganisms and enzymes without significant adverse effects on organoleptic or nutritional properties is feasible (Tajkarimi, Ibrahim, & Cliver, 2010; Tiwari et al., 2009). In the salad supply chain, the control of viable microbial populations is critical to secure food safety and hygiene quality. The contamination and postharvest growth of *Escherichia coli* and *Staphylococcus aureus* originally sourced from animal feces and food machine operator contamination constitute not only a food quality indicator but a risk that could cause an outbreak of food poisoning. Thus, in this study, because both thymol and resveratrol possess potent antimicrobial activities and are suitable potential food ingredients, the antimicrobial activities of thymol and resveratrol used alone or combined against strains of *E. coli* and *S. aureus* were investigated. Applications of thymol and resveratrol in controlling total viable microbial counts of alfalfa sprouts and mushroom slices to guarantee the quality of fresh produce were extended.

## 2 Materials and Methods

### 2.1 Antibacterial reagents, chemicals, and test microorganisms

Thymol was purchased from Sigma Chemical Co. (St. Louis, MO) and resveratrol (with approximately 98% purity) was provided by Glory Biotech Co. (Chiayi, Taiwan). Alfalfa sprouts and fresh mushrooms were purchased from a local supermarket (Chiayi, Taiwan).

Two G(−) bacteria, *E. coli* BCRC 10675 and *E. coli* O157:H7 BCRC 15374, and two G(+) bacteria, *S. aureus* BCRC 12655 and *S. aureus* 10780, were ordered from the Bioresource Collection and Research Center (BCRC) of the Food Industry Research and Development Institute (Hsinchu, Taiwan). Tryptic soy broth (TSB), tryptic soy agar (TSA), and plate count agar (PCA) used for antibacterial activity assessment and total viable count (TVC) enumeration were purchased from Merck (Darmstadt, Germany).

### 2.2 Antibacterial activity determination by paper disk diffusion assay

Thymol and resveratrol dissolved and diluted with 95% ethanol to 20,000 ppm (μg/mL) were prepared and used as source solutions. Ethanol solution (95%) was used as a control. Each test bacterium was precultivated with TSB at 37°C overnight (16–18 hr), and 0.1 mL of the broth was spread onto a prepoured Petri dish containing TSA. Eight‐millimeter (diameter) aseptic paper disks were impregnated with 20 μL of each prepared source solution. The disks were placed onto the TSA plates with the previous bacterial spread and incubated at 37°C for 16–18 hr. Antibacterial activity was evaluated by measuring the diameter of the inhibition zone (DIZ).

### 2.3 Antibacterial activity determination by minimum inhibitory concentration assay

In each well of a 96‐well microplate, 100 μL of TSB was aseptically deposited. Thymol and resveratrol were dissolved in 95% ethanol at 50,000 ppm (μg/mL) and used as source solutions. From each of the source solutions, 100 μL was withdrawn and deposited into a well containing 100 μL of TSB. After pumping using a pipette for well mixing, 100 μL of the mixed solution was withdrawn and deposited into the next well containing 100 μL of TSB and was well mixed. This was repeated stepwise to the 11th well. From the 11th well, 100 μL of the mixed solution was withdrawn and discarded to maintain an even volume among test wells. The 12th well containing 100 μL of TSB was prepared as a control.

For inoculation, each of the 16 hr‐cultivated TSB cultures of the test bacterial strains was serially diluted with TSB to a final microbial population of approximately 10^6^ CFU/mL, from which 50 μl was withdrawn and deposited into each well of a 96‐well microplate containing 100 μL of TSB that contained thymol or resveratrol in various concentrations. The microplates were covered with a lid and were cultivated at 37°C for 24 hr. Turbidity, to indicate bacterial growth at 610 nm of each well, was measured spectrophotometrically using an ELISA reader equipped with the Gen5 Data Analysis Software interface (Epoch Microplate Spectrophotometer, BioTek Instruments Inc., Winooski, VT). The lowest concentration for no growth for each test microorganism was defined as the minimum inhibitory concentration (MIC).

The concentrations of thymol or resveratrol from the first to the 12th wells were estimated to be 16,667, 8333, 4167, 2083, 1042, 521, 260, 130, 65, 32, 16, and 0 ppm, respectively. The correlated ethanol concentrations after serial dilutions from the original thymol or resveratrol dissolved in 95% ethanol were 31.7, 15.8, 7.9, 3.95, 1.98, 1.0, 0.5, 0.25, 0.12, 0.06, 0.03, and 0%, respectively.

### 2.4 Surviving bacterial population enumeration

The 16 hr‐cultivated TSB culture (approximately 10^9^ CFU/mL) for each of the test bacteria was diluted with a 0.1% peptone solution to reach a cell population of 10^6^ CFU/mL and used as an inoculum. Series of 95% ethanol solutions containing specified concentrations of thymol or resveratrol were prepared. Through a series of test tubes, each containing 8.9 mL of sterilized TSB, 1 mL of the prepared thymol or resveratrol solution dissolved in 95% ethanol was replenished and then inoculated with 0.1 mL of the bacterial inoculum and vortexed for well mixing. The final thymol and resveratrol concentrations were 0, 125, 250, and 500 ppm (μg/mL), and the cell population and ethanol concentration were approximately 10^4^ CFU/mL and 9.5%, respectively. After the cell suspension was held at the ambient temperature (24–28°C) for 1, 5, 10, 20, or 40 min, 1 mL from each of the test tubes was withdrawn and diluted with 9 mL of 0.1% peptone solution. From each diluted suspension, 0.1 mL was withdrawn and deposited in a Petri dish and well mixed with the poured melted TSA at approximately 50°C. After agar solidification, the plates were incubated at 37°C for 24 hr, followed by a plate count of colonies (expressed by CFU).

### 2.5 TVC control of alfalfa sprouts and mushroom slices

A series of 15% ethanol aqueous solutions containing various concentrations of thymol or resveratrol at 0, 125, 250, and 500 ppm (w/v) were prepared. Fresh alfalfa sprouts were distributed into a series of plastic cups (each containing approximately 5 g). Fresh mushrooms (*Agaricus bisporus*) were cut into slices approximately 0.3–0.4 cm thick and distributed into a series of plastic cups (each containing approximately 5 g). For TVC control, each cup containing sprouts or mushroom slices was soaked with 100 mL of reverse osmosis (RO) water, 15% ethanol, and 15% ethanol containing various concentrations of thymol or resveratrol for 30 min and drained. Then, the sprouts or slices were rinsed using RO water for 0.5 min, drained, lid‐covered, and stored in a refrigerator at 4°C for 48 hr.

During storage, samples of sprouts or slices were taken at 0, 24, and 48 hr, and each was deposited into a sterile plastic bag, replenished with 45 mL of sterilized 0.1% peptone solution, and homogenized with a Stomacher (Lab‐Blender 80, Sward Medical, London, England) for 2* *min. Aliquots of the homogenate were subjected to serial dilution with 0.1% peptone solution. From each of the solutions after appropriate dilution, 0.1 mL was withdrawn and deposited into a Petri dish and well mixed with approximately 10 mL of melted PCA. After agar solidification, the plates were incubated at 37°C for 48 hr for TVC enumeration. Antibacterial potency as evidenced by viable microbial control was expressed as a log reduction (LR) on the basis of the log value changes of the enumerated TVCs during storage.

### 2.6 Statistical analysis

Triplicate experiments were conducted and measured values were expressed as the mean ± standard deviation. Analysis of variance was conducted using SPSS (Chicago, IL).

## 3 Results and Discussion

### 3.1 Antibacterial activities of thymol and resveratrol

The diameter measurements of the inhibition zone around each specified paper disk impregnated with 20 μL of 95% ethanol, thymol, or resveratrol (20,000 ppm, w/v in 95% ethanol) are shown in Table 1. Thymol and resveratrol were clearly effective in inhibiting growth of the test *E. coli* and *S. aureus* strains. Slight or no inhibitory activity was observed for the disks containing solely 95% ethanol. In comparison, thymol was more potent than was resveratrol in inhibiting growth of the test bacteria (*p* < 0.05).

**Table 1:** Antibacterial activities of thymol and resveratrol dissolved in 95% ethanol against growth of strains of *Escherichia coli* and *Staphylococcus aureus* detected by the disk diffusion test

Antibacterial reagent*	Test bacterial strains and diameter of inhibition zone (DIZ, mm)#			
	*E. coli* BCRC10675	*E. coli* O157:H7 BCRC 15374	*S. aureus* BCRC12655	*S. aureus* BCRC10780
Thymol	17.9 ± 2.8^a^	23.9 ± 2.8^a^	22.8 ± 1.3^a^	21.9 ± 1.3^a^
Resveratrol	17.3 ± 1.3^a^	18.5 ± 1.0^b^	17.5 ± 0.6^b^	19.5 ± 0.6^a^
95% ethanol	9.2 ± 1.1^b^	9.9 ± 1.9^c^	10.9 ± 0.9^c^	11.8 ± 2.2^b^

*Resveratrol and thymol: 20,000 ppm (μg/mL, in 95% ethanol).

#Diameter of inhibition zone >8 mm.

#Each value represents mean ± SD (*n* = 4). Data with different letters in the same column indicate statistically significant differences between antibacterial reagents at *p* < 0.05.

The MIC of thymol and resveratrol ranged from 16 to 166,667 ppm (ethanol concentration ranged from 0.03% to 31.7%) (Table 2). The MIC of thymol against growth of the test bacteria was 65–130 ppm (ethanol concentration ranged from 0.12% to 0.25%). The MIC of resveratrol against growth of *E. coli* was 521 ppm (ethanol concentration was 1.0%) and 130–260 ppm against *S. aureus* (ethanol concentration ranged from 0.25% to 0.5%). Ethanol (95%) was used as a solvent in source solution preparation. The MIC of ethanol was between 3.95% and 7.9%.

**Table 2:** Minimum inhibitory concentration (MIC) of thymol and resveratrol against growth of the test strains of *Escherichia coli* and *Staphylococcus aureus*

Antibacterial ingredient	Test bacterial strains and MIC			
	*E. coli* BCRC 10675	*E. coli* O157:H7 BCRC15374	*S. aureus* BCRC12655	*S. aureus* BCRC10780
Thymol (ppm)	65–130	65–130	65–130	65–130
Resveratrol (ppm)	521	521	130–260	130–260
Ethanol (%, v/v)	3.95–7.90	3.95–7.90	3.95–7.90	3.95–7.90

In a previous study, the DIZs of thymol against growth of *S. aureus* and *E. coli* were 25 ± 0.98 and 13 ± 0.51 mm and the MIC values were 62.5 and 250 ppm, respectively (Mathela, Singh, & Gupta, 2010). In another study, the minimum bactericidal concentration of thymol ranged from 225 to 450 ppm for *E. coli* and was 225 ppm for *S. aureus* (Cosentino et al., 1999). For resveratrol, the MICs against growth of *S. aureus* and *E. coli* were 100–200 and >400 ppm (dissolved in DMSO), respectively (Paulo et al., 2010). The inhibitory concentration (IC_100_) of resveratrol dissolved in ethanol against growth of *S. aureus* and *E. coli* was >200 ppm (Docherty, Fu, & Tsai, 2001). The MIC of resveratrol against growth of *S. aureus* was 171 ppm (dissolved in 1.7% DMSO) (Chan, 2002). When ethanol or DMSO was used as a solvent to increase ingredient solubility, higher solvent concentrations resulted in higher antibacterial activity (Chan, 2002; Filip et al., 2003). In this study, the MIC of thymol against growth of the test G(+) *S. aureus* and G(−) *E. coli* strains varied in a limited range (Table 2). This is in agreement with the report that thymol or thymol containing essential oils exhibits a similar inhibitory effect against growth of *S. aureus* and *E. coli* (Cosentino et al., 1999; Lv, Liang, Yuan, & Li, 2011). Another study reported that thymol is more effective for inhibiting growth of G(+) *S. aureus* than is G(−) *E. coli* (Mathela et al., 2010). In this study, resveratrol was more effective in inhibiting growth of G(+) *S. aureus* than that of G(−) *E. coli* (Table 2). This is in agreement with the observations reported by Paulo et al. (2010). Apparently, the observed antibacterial activities varied depending upon the nature of the test microorganism, chemical structure, and used solvent.

MIC values for ethanol were 3.95% to 7.9% (Table 2). Viable *E. coli* and *S. aureus* cells suspended in nutrient broth containing 12.5%, 20%, or 40% ethanol combined with NaCl decreased with an increase in the ethanol concentration and time of treatment (Huang, Weng, & Chiou, 2001). When the cells were treated with 40% ethanol combined with ≥0% NaCl, all test strains lost viability within 5 min. In this study, ethanol was used in stock to source solution preparation of the test agents, and its concentration was diluted serially with TSB; the antibacterial activity of ethanol (3.95%) should be considered for combining the test antibacterial ingredients. Particularly for the tests with an MIC higher than 2,000 ppm, the copresent solvent of ethanol influencing microbial responses of growth is likely. The varying effective doses between laboratories may be related to the solvent used and the varying resistance of the test bacteria.

### 3.2 Survival test of *E. coli* and *S. aureus*

**Figure 1:**
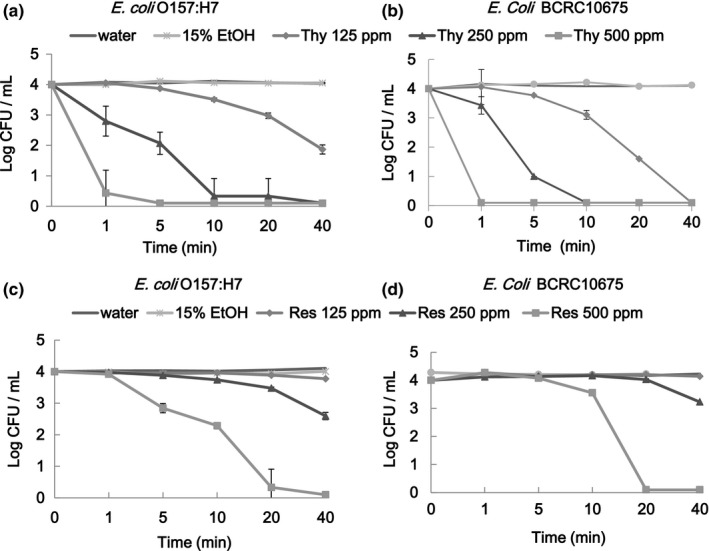
Surviving microbial populations of *Escherichia coli* O157:H7 BCRC 15374 and *E. coli* BCRC 10675 subjected to treatment with thymol (Thy) (a and b) and resveratrol (Res) (c and d) for various intervals

**Figure 2:**
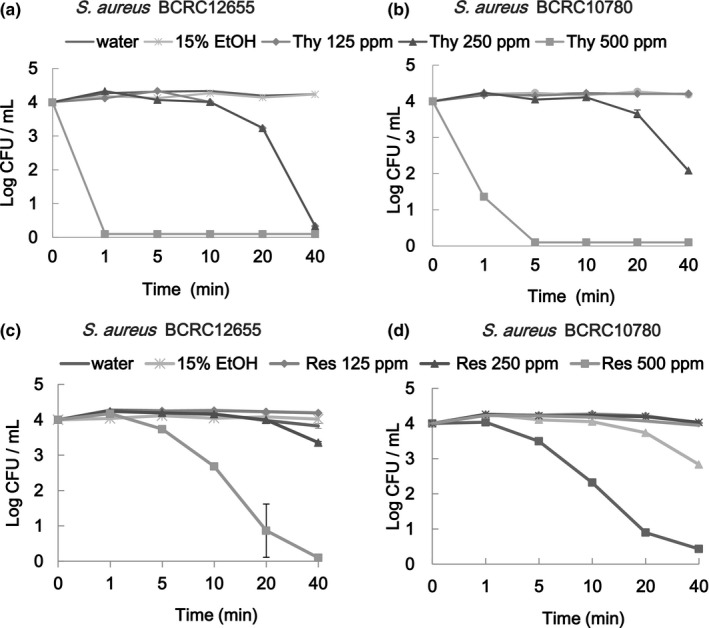
Surviving microbial populations of *Staphylococcus aureus* BCRC 12655 and *S. aureus* BCRC 10780 subjected to treatments with thymol (Thy) (a and b) and resveratrol (Res) (c and d) for various intervals

In the survival test, *E. coli* BCRC 10675, *E. coli* O157:H7 BCRC 15374, *S. aureus* BCRC 12655, and *S. aureus* BCRC10780 cells were suspended in TSB containing various concentrations of thymol or resveratrol for specified intervals prior to collecting aliquots for microbial population enumeration (Figures 1 and 2). Microbial populations for both bacteria decreased with an increase in ingredient dose and the duration of treatment. At the same concentration level, thymol was more effective than was resveratrol in exhibiting antibacterial activity. No viable growth was detected for the test bacteria suspended in TSB containing 500 ppm thymol for 1–5 min. *E. coli* cells reached 3LR relative to the initial population after treatment with thymol at 250 ppm for 10 min (Figure 1a and b) or treatment with resveratrol at 500 ppm for 20 min (Figure 1c and d). For *S. aureus*, 3LR was achieved through treatment with thymol at 500 ppm for 1–5 min or at 250 ppm for >40 min (Figure 2a and b) or treatment with resveratrol at 500 ppm for 20 min (Figure 2c and d). This is in agreement with the report of Burt & Reinders (2003), who used oregano essential oil (containing thymol as a major component) to inhibit the growth of *E. coli* O157:H7 and observed killing times of 1 min at 625 ppm and 5 min at 156 and 312 ppm as well as a time to achieve >2LR of 5 min at 78 ppm. Thymol at 250 ppm was capable of achieving complete growth inhibition (>4LR) of *S. aureus* and *E. coli* through 40 and 10 min of treatment, respectively. In this study, for resveratrol at 500 ppm, achieving complete growth inhibition of *S. aureus* and *E. coli* required 40 and 20 min, respectively. This is not in agreement with the general observation that essential oils are more effective at inhibiting growth of G(+) bacteria than that of G(−) bacteria (Burt, 2004; Cosentino et al., 1999; Lv et al., 2011). The difference might be attributable to direct contact of the cells in the TSB suspension containing ethanol and specific cell membrane permeability circumstances leading to a bactericidal rather than bacteriostatic effect.

Cell wall structural differences between G(+) and G(−) bacteria in relation to varying susceptibility to antibacterial agents have been extensively investigated (Burt, 2004; Cosentino et al., 1999; Lambert, Skandamis, Coote, & Nychas, 2001; Mathela et al., 2010). For thymol interaction with *E. coli* cells (Xu et al., 2008), in addition to its lipophilic structure that enhances membrane permeability, the hydrophilic nature of the –OH moiety may interrupt and depolarize the cytoplasmic membrane. Carvacrol and thymol may cause disintegration of the outer membrane of G(−) bacteria, releasing lipopolysaccharides and increasing the permeability of the cytoplasmic membrane to ATP (Burt, 2004). For resveratrol used to inhibit growth of *Bacillus cereus* (Paulo et al., 2010), morphological alteration and cellular growth arrest were observed through SEM and cell cycle examination. Thus Gram stain‐differentiated structural properties along with the nature of thymol and resveratrol must respond to various bacterial and chemical interactions is without doubt.

In comparison with the detected MIC and survival concentrations (Table 2, Figures 1 and 2), the survival concentrations for thymol were two‐ to fourfold higher than the MIC to inhibit growth of *E. coli* and four‐ to eightfold higher than the MIC to inhibit growth of *S. aureus*. In the survival (bactericidal) test, cells suspended in TSB containing 9.5% ethanol and either thymol or resveratrol must be killed, be wounded (injury), or survive. In general, only the surviving (resistant) cells could be recovered. A similar observation in which resveratrol was used to treat *Bacillus cereus* was reported (Paulo et al., 2010). The MIC test mostly addresses bacteriostatic rather than bactericidal activity.

**Figure 3:**
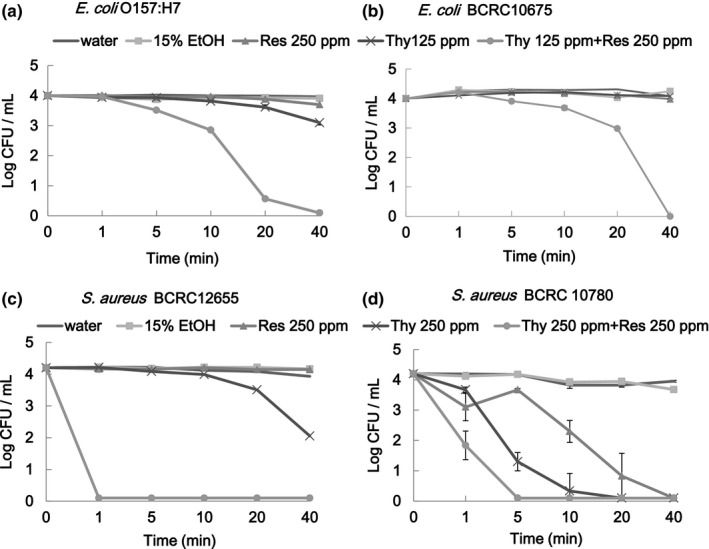
Surviving microbial populations of *Escherichia coli* O157:H7 (a), *E. coli* BCRC10780 (b), *Staphylococcus aureus* BCRC 12655 (c), and *S. aureus* BCRC 10780 (d) subjected to treatments with thymol (Thy) combined with resveratrol (Res) for various intervals

To address the synergistic antibacterial effect, *E. coli* cells were treated with 15% ethanol, thymol at 125 ppm, resveratrol at 250 ppm, or thymol at 125 ppm combined with resveratrol at 250 ppm for 0–40 min (Figure 3a and b). *S. aureus* cells were treated with 15% ethanol, thymol at 250 ppm, resveratrol at 250 ppm, or thymol at 250 ppm combined with resveratrol at 250 ppm for 0–40 min (Figure 3c and d). The time for *E. coli* O157:H7 and *E. coli* BCRC 10675 cells to achieve 3LR through combined treatment with thymol and resveratrol was <20 and 40 min, respectively. For the other treatments, times to achieve 3LR were longer than 40 min. For *S. aureus* BCRC12655 cells, the 3LR time was >40 min for the treatment with thymol at 250 ppm and less than 1 min for combined treatment with thymol at 250 ppm and resveratrol at 250 ppm (Figure 3c). For *S. aureus* BCRC10780 cells, 3LR was achieved at 10 min for the treatment with thymol at 250 ppm and in <5 min for the combined treatment with thymol at 250 ppm and resveratrol at 250 ppm. A synergistic effect of thymol and resveratrol against growth of *S. aureus* BCRC12655 was clearly observed (Figure 3c). The synergistic effect being more effective in inhibiting growth of *S. aureus* than that of *E. coli* is in agreement with the reported observation of Lv et al. (2011).

### 3.3 TVC control of alfalfa sprouts and mushroom slices

Alfalfa sprouts are commonly served as components of salads in restaurants. The TVC for alfalfa sprouts is generally observed to be higher than 8.5 log CFU/g, which is attributed to the rapid growth of the contaminated microorganisms from seeds and water during cultivation. Such contamination may cause outbreaks of food poisoning if human pathogenic microorganisms are present (Abadias, Usall, Anguera, Solsona, & Vinas, 2008; Beuchat, Ward, & Pettigrew, 2001; Taormina & Beuchat, 1999). In addition to improving cultivation circumstances and related practices to minimize the initial viable microbial population, TVC control through treatment with antibacterial ingredients after harvest along with cold chain storage, delivery, and consumption deserve intensive investigation to ensure optimal hygiene quality.

**Figure 4:**
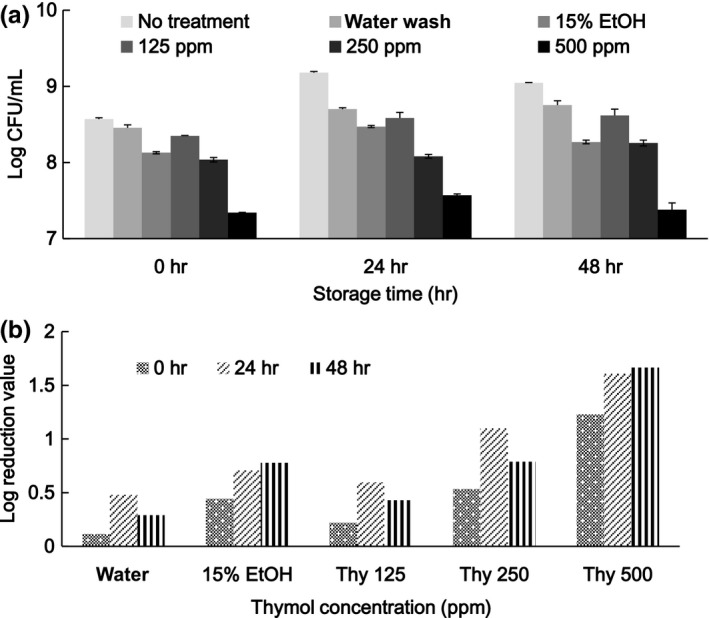
Microbial populations (a) and log reduction (b) of alfalfa sprouts subjected to treatments with thymol (Thy) at various concentrations and storage at 4°C for 48 hr

Fresh alfalfa sprouts were soaked with 15% ethanol alone and various concentrations (0–500 ppm) of thymol dissolved in 15% ethanol for 30 min, followed by refrigerator storage, and log values of the TVC for the samples were obtained after 24 and 48 hr of storage (Figure 4). The initial log TVC was higher than 8.5 log CFU/g. This is in agreement with the populations detected in other laboratories (Abadias et al., 2008; Beuchat et al., 2001; Taormina & Beuchat, 1999). The TVC of the control increased with an increase in refrigerator storage time. LR values for treatment with water washing were 0.48 and 0.29 after 24 and 48 hr of storage, respectively. The LR values were 0.71 and 0.78 for treatment with 15% ethanol after 24 and 48 hr of storage, respectively. Apparently, 15% ethanol treatment was effective in TVC reduction. The initial TVC was reduced immediately after treatment and, during storage, the effectiveness was further enhanced in a dose‐dependent manner using the dissolved thymol. Soaking with 500 ppm thymol was most effective at ensuring a limited TVC change during 48 hr of storage. The effectiveness of thymol in TVC control of alfalfa seeds was also reported by Beuchat et al. (2001).

**Figure 5:**
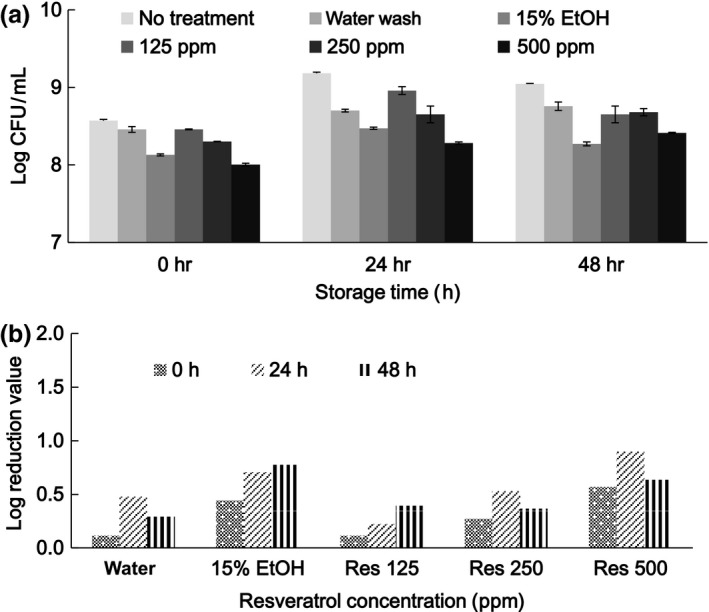
Microbial populations (a) and log reduction (b) of alfalfa sprouts subjected to treatments with resveratrol (Res) at various concentrations and storage at 4°C for 48 hr

TVC changes for alfalfa sprouts after treatment with resveratrol and storage in a refrigerator for 48 hr are shown in Figure 5. The initial TVC values decreased in a resveratrol concentration‐dependent manner. The initial LR ranged from 0.11 to 0.57 for treatments with water washing, 15% ethanol, and 15% ethanol combined with resveratrol supplementation at 125, 250, and 500 ppm. Treatment with resveratrol at 500 ppm was effective for achieving 0.90 LR after 24 hr of storage. In comparison with treatment with 15% ethanol, no further synergistic effect was achieved through supplementation with resveratrol at 125 and 250 ppm. Resveratrol is an active biological polyphenol and, at low concentrations, it may play a role in the protection of viable or injured microbes.

**Figure 6:**
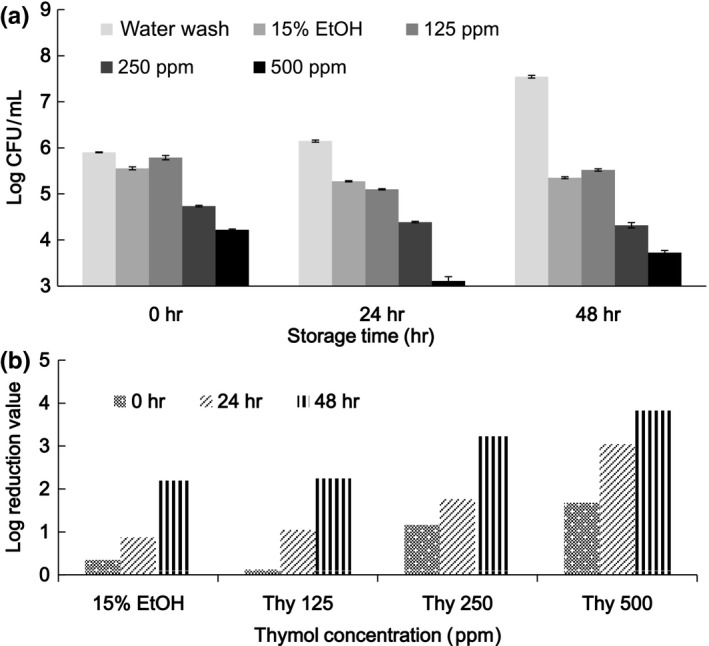
Microbial populations (a) and log reduction (b) of mushroom slices subjected to treatments with thymol (Thy) at various concentrations and storage at 4°C for 48 hr

**Figure 7:**
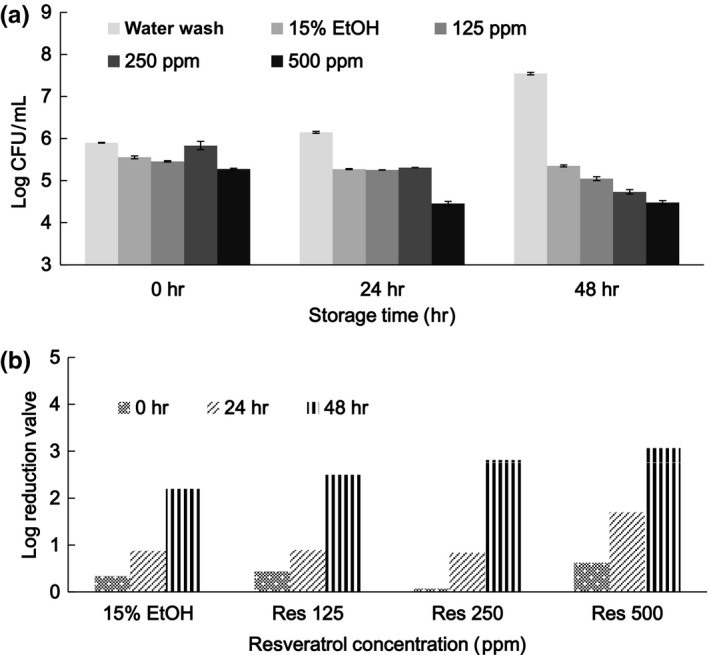
Microbial populations (a) and log reduction (b) of mushroom slices subjected to treatments with resveratrol (Res) at various concentrations and storage at 4°C for 48 hr.

For fresh mushrooms sliced and treated with water washing, 15% ethanol, and 15% ethanol solution containing various concentrations of thymol prior to refrigerator storage for 48 hr, the initial TVC values and changes during storage are shown in Figure 6. Initial TVC values expressed by LR decreased from 0.11 to 1.68 in a dose‐dependent manner from 0 to 500 ppm. During storage for 48 hr, TVC values for water washing increased from 6 to 8 log CFU/g. This may be attributed to nutrients released from the cut (wound) surface of mushroom slices that are supportive to microbial growth. When the slices were soaked with 15% ethanol and after 24 and 48 hr of storage, the LR values were 0.87 and 2.2, respectively. In comparison with values achieved through water washing, a synergistic effect between 15% ethanol and thymol supplementation was noticed. After water wash treatment and 48 hr of storage, LR values for 250 and 500 ppm thymol treatments were 3.2 and 3.8, respectively. When resveratrol was replaced with thymol and subjected to similar treatments (Figure 7), 15% ethanol was effective in decreasing the initial TVC, and an enhanced effect of resveratrol in decreasing the initial TVC was observed when it was added at concentrations up to 500 ppm. During storage for 48 hr, in comparison with water washing, resveratrol at a high concentration was more effective at decreasing the TVC. The LR for the treatment with resveratrol at 500 ppm was 3.06.

The antimicrobial activity of thymol was more effective than was that of resveratrol. Thymol at >500 ppm was effective for alfalfa sprouts and thymol at >250 ppm was effective for mushroom slices. A previous study reported that thymol at 0.4 mg/mL (400 ppm in 4% ethanol) was effective at inhibiting growth of surface‐inoculated *Salmonella* on grape tomatoes (Lu & Wu, 2010). Treatment with thymol at 200 ppm combined with 2%–5% SDS and 2000 ppm acetic acid was reported to result in an equivalent antibacterial effectiveness (>6.9 LR) to 400 ppm thymol or 200 ppm chlorine solution (Lu et al., 2014). After soaking tomatoes in solution containing 100–200 ppm oregano essential oil and storing them at 25°C for 12 days or at 4°C for 1 month, effective mold growth control was achieved only through storage at 4°C (Ibrahim & Al‐Ebady, 2014).

## 4 Conclusion

Thymol and resveratrol were both effective in inhibiting growth of the test *E. coli* and *S. aureus* strains, and thymol was more effective than was resveratrol. Our results demonstrate that thymol in combination with resveratrol exerted a synergistic antibacterial effect in inhibiting growth of *E. coli* and *S. aureus*. When alfalfa sprouts were treated with 500 ppm thymol or 500 ppm resveratrol for 30 min and stored at 4°C for 48 hr, the LRs of the TVC were >1.7 and 0.63, respectively. For mushroom slices subjected to a similar treatment, the LRs were >3.0. Resveratrol and thymol are both novel bioactive phytochemicals and confer perspective potency in the development of the related value‐added products. In addition to TVC control, the health‐enhancing benefits of resveratrol in addition to its antibacterial activity must be demonstrated. In the future, investigations could be extended to sensory and functional property characterization and related product development.

## Conflict of Interest

None declared.
